# The Precautionary Principle: Dolan and Rowley Respond

**DOI:** 10.1289/ehp.0901370R

**Published:** 2010-01

**Authors:** Mike Dolan, Jack Rowley

**Affiliations:** Mobile Operators Association, London, United Kingdom, E-mail: mikedolan@ukmoa.org; GSM Association, London, United Kingdom, E-mail: jrowley@gsm.org

We thank Zinelis for his interest in our article (Dolan and Rowley 2009). However, it appears from his comments on the recommendations of the International Commission on Non-Ionizing Radiation Protection (ICNIRP), that he misunderstands the scientific basis and scope of the evidence used to establish those exposure guidelines. The ICNIRP (1998) stated clearly that for the frequencies relevant to mobile communications the restrictions are “provided to prevent whole-body heat stress and excessive localized tissue heating.” This is based on evidence of established health effects. In respect to claims of effects from low-level and modulated exposures, the ICNIRP (1998) stated that

Overall, the literature on athermal effects of AM [amplitude modulated] electromagnetic fields is so complex, the validity of reported effects so poorly established, and the relevance of the effects to human health is so uncertain, that it is impossible to use this body of information as a basis for setting limits on human exposure to these fields.

The ICNIRP keeps the scientific evidence under review and recently restated that the 1998 recommendations remain valid (ICNIRP 2009), again noting in respect of claims of nonthermal affects that

With regard to non-thermal interactions, it is in principle impossible to disprove their possible existence but the plausibility of the various non-thermal mechanisms that have been proposed is very low.

Zinelis makes an analogy with risks from asbestos; however, this is flawed. By way of example, animal studies show evidence of harm from exposure to asbestos (International Agency for Research on Cancer 1987), whereas in respect to radiofrequency exposures, the animal studies consistently show that carcinogenic effects are not likely, even at exposure levels above those from mobile telephones (Scientific Committee on Emerging and Newly Identified Health Risks 2009).

We do accept the involuntary nature of exposure to radio signals from base stations; this in integral to providing the mobile phone services that almost 4 billion people voluntarily use and is a matter for risk perception, not risk assessment. We conclude by reiterating that the precautionary principle cannot be used to justify measures to restrict radio frequency exposures from mobile phones or base stations when there is no scientifically plausible evidence of a hazard to human health.
